# Deaf adult roles: recommendations from family-centered early intervention

**DOI:** 10.1093/jdsade/enag031

**Published:** 2026-06-05

**Authors:** Elaine Gale

**Affiliations:** Department of Special Education, Hunter College of the City University of New York, New York, United States

## What is the issue?

Deaf adults have valuable cultural and linguistic expertise rooted in lived experience. This expertise contributes to early intervention programs in many ways across diverse roles. Examples include partnering with families, serving as role models, supporting early sign language access and advocating for rights. These roles also broaden cultural perspectives and strengthen professional collaboration. Together, lived experience and expertise, along with relevant competencies, contribute to the overall development and well-being of Deaf children. In this article, Deaf is used inclusively across diverse hearing levels, technology use and identities.

Recognizing the value of lived experience and expertise, the 2013 Family-Centered Early Intervention (FCEI) principles included recommendations related to Deaf adult roles in two of ten principles. However, this inclusion may not have fully reflected the range and importance of these roles because they continue to be inconsistent or nonexistent. This highlights the need to expand and consistently integrate these roles throughout FCEI principles.

## Why is it important?

When Deaf adult roles are limited or not emphasized, early intervention programs risk defaulting to a medicalized approach. This reduces opportunities for families to receive time-sensitive information and resources that effectively support their Deaf children. For example, many people may not realize that relying primarily on spoken language, even with hearing technology, is not always fully or easily accessible to Deaf children and that their language, developmental, and mental health outcomes remain highly variable. They may not know that natural sign languages and bimodal bilingual approaches not only provide accessible language from the start but also support spoken language development. Strong early language foundations are critical because they support cognitive, social–emotional, and identity development while reducing the risk of language deprivation.

## How can this support Deaf children, families, and professionals?

The impact of Deaf adult roles within early intervention extends across Deaf children, families, and professionals in distinct yet interconnected ways. Explicitly embedding these roles across all FCEI principles highlights this importance.

For Deaf children, interacting with multiple Deaf adults early in life is especially important because this period is critical for language and identity development. Deaf adults teach sign language, model visual communication strategies, and create language-rich environments. These interactions also support identity formation, expand children’s sense of belonging, and strengthen connection to the Deaf community.

For families, meeting diverse Deaf adults early is significant because over 90% of Deaf babies have hearing parents. In many cases, their child is the first Deaf person they meet. When families have early opportunities to connect with Deaf adults across varied roles, they gain timely access to lived experience, broaden perspectives, envision future possibilities, and build confidence in raising their Deaf child from an early age.

For professionals, working with a range of Deaf adults is essential because it strengthens collaborative practice and brings perspectives grounded in lived experience to the team. These perspectives deepen understanding beyond a solely medical approach by emphasizing equitable language access, identity, and overall development.

### What has been done?

In 2018, to better emphasize the value of Deaf adult roles in early intervention, participants in a FCEI Deaf Leadership pre-conference workshop reviewed the 2013 FCEI principles. Through this process, the workshop group began updating principles with recommendations that more explicitly referenced these roles to strengthen support for Deaf children and their stakeholders.

**Table 1:** Deaf adult role recommendations for EI providers.

**EI Provider actions**
Foundation Principles
**Principle 1 Early intervention following identification: Early, timely, and equitable provision of supports**
P1.6. Invite families to connect with DHH adult-to-family supports that include opportunities to meet a variety of trained DHH adults and professionals with varying hearing levels and lived experiences; provide information about benefits of DHH adult supports; provide access to information that builds understanding about what it means to be DHH
**Principle 2 Family–EI Provider relationships: Partnerships, engagement, capacity building and reflection**
P2.8 Provide families with information about a range of community partners and supports, including DHH adult-to-family and family-to-family connections
Support Principles
**Principle 3 Family support: Basic needs, strengths/challenges, and connections**
P3.12 Share information in response to family questions and needs about opportunities for DHH adult-to-family support, identify resources for making DHH adult-to-family connections, and assist families in making connections to a variety of DHH adults or DHH adult-to-family supports
**Principle 4 Child well-being: Infant/child development, positive social–emotional functioning, child welfare, and safeguarding**
P4.9 Provide information in response to family questions about structured opportunities for the child who is DHH to engage with other children who are DHH (e.g., play groups, learning opportunities, community activities) and interact with a variety of DHH adults to promote positive social–emotional functioning
**Principle 5 Language and communication: Early and consistent access, approaches and opportunities, and language-rich environments**
P5.12 Provide families access to opportunities to engage with a range of adults who are DHH (including those who communicate through spoken language, sign language, cued speech/cued language, or who utilize a variety of approaches) who can teach families about language opportunities and communication approaches, and who can share their lived experiences with communication and technologies P5.15 Gain knowledge about working with families, child development, and working with young children (for all members of FCEI teams, including DHH and family leaders and professionals that collaborate with the team)
**Principle 6 Family decisions: Decision-makers, culture and context, information, and adaptable decisions**
P6.12 Provide families with opportunities to engage with adults and older children who are DHH with diverse lived experiences to support informed decision-making
Structure Principles
**Principle 7 Trained FCEI-DHH Providers: Dispositions and competencies**
P7.9 Gain understanding of the value of including on the FCEI-DHH team individuals who have acquired the necessary competencies for engaging in FCEI-DHH and who possess relevant lived experience, including family leaders and DHH adults
P7.12 Share knowledge about EI-specific competencies with family leaders and individuals who are DHH to build supports; gain knowledge from family leaders and a diversity of DHH adults to obtain valuable insights from their lived experiences to build own competencies
**Principle 8 Collaborative teamwork among professionals: Composition, responsibilities, and coordination of teams**
P8.9 Gain and share knowledge with families in response to their questions about the benefits and roles of professionals who partner with collaborative teams (e.g., family-to-family support, DHH adult-to-family support, interpreters, cultural brokers, community health workers) and partner with these professionals
**Principle 9 Developmental assessment: Purpose, approaches, skilled assessors, and interventions**
P9.13 Collaborate with trained, competent professionals who are DHH in the assessment process, when available, as they may be able to provide unique insights on visual communication strategies as well as children’s language use and communicative attempts, and family behaviors (e.g., family-child interaction, social–emotional development, visual strategy use)
**Principle 10 Progress monitoring: Relevance, effectiveness, and tracking FCEI-DHH program/service and system outcomes**
P10.4 Provide information for families about the roles of various EI Providers and their involvement in program monitoring; provide information about DHH adults and family leaders involved in supporting the family (whether they are members of the FCEI-DHH team or partnering with the team); ensure that families understand that data are aggregated and that privacy is protected

*Note*. FCEI-DHH = Family-Centered Early Intervention Deaf/Hard of Hearing; EI = Early Intervention. DHH is used throughout the table to match terminology in the 2024 FCEI-DHH special issue (Sass-Lehrer, 2024). This table presents recommendations excerpted from the special issue that explicitly reference Deaf adult roles. Additional relevant recommendations not listed here also apply, even when Deaf adults are not directly mentioned. See the special issue for more information.

In 2019, the Deaf Leadership International Alliance (DLIA) was established to advocate for Deaf adult lived experience and expertise throughout early intervention programs. Many participants from the 2018 pre-conference workshop became DLIA members and continued developing the recommendations for each principle. Also in 2019, DLIA, the FCEI Executive Group, and the Global Parents of Deaf and Hard of Hearing collaborated to revise the 2013 principles from multiple perspectives. As a result, the expanded FCEI principles incorporated broader international and cultural perspectives, updated empirical evidence, and included human development research.

In 2024, the expanded FCEI principles were published in a special issue with multiple recommendations referencing Deaf adult roles across all ten principles.

### What can be done?

To enhance usability and support implementation, this article organizes the 2024 FCEI recommendations that explicitly reference Deaf adult roles into three tables: provider recommendations (Table 1), family recommendations (Table 2), and program/service and system recommendations (Table 3). Families and professionals can use these tables to access and translate recommendations into practical strategies and concrete actions that benefit Deaf children. For example, families can use the tables to build awareness and advocate for services, while professionals can use them to strengthen practice and implement program changes.

**Table 2:** Deaf adult role recommendations for families.

**Family activities and outcomes**
Foundation Principles
**Principle 1 Early intervention following identification: Early, timely, and equitable provision of supports**
P1.6. Consider opportunities for and benefits of engaging with DHH adult-to-family supports in a timely and culturally appropriate manner (e.g., to support adjustment to having a child who is DHH and to understand what it means for their child and their family)
**Principle 2 Family–EI Provider relationships: Partnerships, engagement, capacity building and reflection**
P2.8 Engage with community partners, including DHH adult-to-family and family-to- family connections, as well as family-identified community supports (e.g., religious/spiritual groups, other community groups)
Support Principles
**Principle 3 Family support: Basic needs, strengths/challenges, and connections**
P3.12 Consider engaging in opportunities for DHH adult-to-family support and DHH community supports; consider meeting with DHH adults with a variety of lived experiences
**Principle 4 Child well-being: Infant/child development, positive social–emotional functioning, child welfare, and safeguarding**
P4.9 Ask questions and gain knowledge about available opportunities for, and benefits of, having their child who is DHH interact with other children who are DHH and interact with a variety of DHH adults to support their child’s positive social–emotional functioning
**Principle 5 Language and communication: Early and consistent access, approaches and opportunities, and language-rich environments**
P5.12 Consider opportunities to engage with a variety of adults who are DHH and are trained to work with families, understand child development, and can provide valuable insights into building and expanding communication and language abilities and using various communication approaches and technologies
**Principle 6 Family decisions: Decision-makers, culture and context, information, and adaptable decisions**
P6.12 Engage in opportunities to meet with adults and older children who are DHH with diverse lived experiences to support informed decision-making
Structure Principles
**Principle 7 Trained FCEI-DHH Providers: Dispositions and competencies**
P7.9 Gain understanding of the value of having EI Providers with necessary FCEI-DHH competencies and the value of inclusion of individuals who possess relevant lived experience, including family leaders and DHH adults
**Principle 8 Collaborative teamwork among professionals: Composition, responsibilities, and coordination of teams**
P8.9 Gain understanding about the benefits and roles of professionals who partner with collaborative teams (e.g., family-to-family support, DHH adult-to-family support, interpreters, cultural brokers, community health workers); evaluate the information received in light of family values, strengths, needs, and goals
**Principle 9 Developmental assessment: Purpose, approaches, skilled assessors, and interventions**
P9.13 Consider the value of including professionals who are DHH in assessment and the unique insights they can provide
**Principle 10 Progress monitoring: Relevance, effectiveness, and tracking FCEI-DHH program/service and system outcomes**
No family recommendations within Principle 10 explicitly reference Deaf adults. See the special issue for relevant recommendations.

*Note*. FCEI-DHH = Family-Centered Early Intervention Deaf/Hard of Hearing; EI = Early Intervention. DHH is used throughout the table to match terminology in the 2024 FCEI-DHH special issue (Sass-Lehrer, 2024). This table presents recommendations excerpted from the special issue that explicitly reference Deaf adult roles. Additional relevant recommendations not listed here also apply, even when Deaf adults are not explicitly referenced. See the special issue for more information.

**Table 3:** Deaf adult role recommendations for program/service and system processes.

**Program/service and system processes **
Foundation Principles
**Principle 1 Early intervention following identification: Early, timely, and equitable provision of supports**
P1.10 Create opportunities for ongoing professional development for EI Providers to support families with initiating FCEI-DHH (e.g., helping to navigate appointments and follow-up, referring for/providing additional supports, and introducing family-to-family as well as family-to-DHH adult contacts)
P1.11 Create programmatic opportunities for family-led family-to-family support networks and DHH adult-led DHH adult-to-family support networks as part of the FCEI- DHH system
**Principle 2 Family–EI Provider relationships: Partnerships, engagement, capacity building and reflection**
P2.12 Incorporate professionals/leaders who are DHH in systems and programs to provide guidance to professionals and families about partnership, engagement, and capacity building
P2.14 Collaborate systematically with family-led and DHH-led communities and organizations
Support Principles
**Principle 3 Family support: Basic needs, strengths/challenges, and connections**
P3.7 Provide opportunities for EI Providers to gain knowledge about the benefits of and opportunities for connecting with a variety of DHH adults with diverse backgrounds for DHH adult-to-family support
P3.8 Develop and implement formalized family-to-family and DHH adult-to-family support networks, including families with diverse perspectives and experiences as well as individuals who are DHH with diverse perspectives and experiences
P3.9 Develop mechanisms for remotely connecting families to supports including FCEI- DHH supports, family-to-family supports, and DHH adult-to-family supports, to increase engagement (e.g., deliver FCEI-DHH supports via telepractice; make it possible for EI Provider-family connections to take place via cellphone or video—as appropriate for the context and the families)
P3.11 Incorporate family members, parents, and DHH leadership into the strategic development of FCEI-DHH to bolster family support
**Principle 4 Child well-being: Infant/child development, positive social–emotional functioning, child welfare, and safeguarding**
P4.6 Provide information and promote access for children who are DHH to connect with community-based adults who are DHH, who can serve as adult models of what it may be like to be DHH to promote child self-acceptance and well- being
**Principle 5 Language and communication: Early and consistent access, approaches and opportunities, and language-rich environments**
P5.13 Provide opportunities for key leaders (DHH and hearing professionals, researchers, parents, parent leaders, and DHH community members) to identify ongoing system-wide needs related to language and communication access
**Principle 6 Family decisions: Decision-makers, culture and context, information, and adaptable decisions**
P6.8 Provide families with opportunities to engage with DHH adults with diverse lived experiences to support informed decision-making
Structure Principles
**Principle 7 Trained FCEI-DHH Providers: Dispositions and competencies**
P7.7 Hire, train, and compensate family leaders and DHH adults and ensure that they possess needed competencies to serve as trained and knowledgeable EI Providers
P7.8 Create mechanisms for team development between EI Providers who are DHH and EI Providers who are hearing to improve or ensure cultural understanding and enhance communication as well as interactional skills among all EI Providers
**Principle 8 Collaborative teamwork among professionals: Composition, responsibilities, and coordination of teams**
P8.5 Establish mechanisms that promote EI Providers’ ability to partner with organizations or individuals to address family needs (e.g., system-wide mechanisms for referring to family-to-family support and DHH adult-to-family support, mechanisms to facilitate collaboration with organizations that employ community health workers or cultural brokers, and ways to engage professionals who can support child and family goals yet are not formal members of FCEI-DHH teams)
**Principle 9 Developmental assessment: Purpose, approaches, skilled assessors, and interventions**
P9.3 Support ongoing consultation, training, and educational opportunities for EI Providers (including professionals who are DHH, who may be particularly useful in providing insights about language and communication development, social–emotional development, and visual strategy use) to improve their skills in conducting developmental assessments of children who are DHH
P9.11 Seek advice from family leaders and DHH leaders about issues that should be considered in assessing child and family outcomes
**Principle 10 Progress monitoring: Relevance, effectiveness, and tracking FCEI-DHH program/service and system outcomes**
P10.11 Develop and implement measures to monitor how DHH adults are infused or included in FCEI-DHH programs/services or systems, the quality of the supports offered by DHH adults, and the impact of their involvement on families receiving FCEI-DHH supports
P10.15 Develop plans to address when benchmarks are not met (with the inclusion of relevant invested parties including family leaders, DHH and hearing professionals, and Deaf community members)
P10.16 Design and implement formal and informal monitoring systems for system-wide quality improvement that are informed by key invested parties (e.g., EI Providers, family members and family leaders, DHH adults and DHH leaders, researchers and professionals with expertise related to supporting children who are DHH and their families, cultural brokers, etc.)
P10.17 Develop mechanisms to receive and resolve concerns or complaints from relevant invested parties about EI Providers, DHH adult-to-family and family-to-family supports, and program/service or system provision of FCEI-DHH interventions or supports

*Note*. FCEI-DHH = Family-Centered Early Intervention Deaf/Hard of Hearing; EI = Early Intervention. DHH is used throughout the table to match terminology in the 2024 FCEI-DHH special issue (Sass-Lehrer, 2024). This table presents recommendations excerpted from the special issue that explicitly reference Deaf adult roles. Additional relevant recommendations not listed here also apply, even when Deaf adults are not explicitly referenced. See the special issue for more information.

## Conclusion

Deaf adults play a critical role in strengthening early intervention programs by bringing lived experience and expertise that support Deaf children’s language access, development, and well-being. Expanding these roles across all FCEI principles ensures that Deaf children, families, and professionals benefit from culturally informed, language-rich, and collaborative approaches. The expanded 2024 FCEI principles provide more explicit recommendations for including Deaf adults consistently throughout early intervention programs. The three user-friendly tables in this article offer practical family activities and professional actions that strengthen implementation, broaden perspectives, and improve outcomes for Deaf children. For this article, three user-friendly tables were created to organize and highlight these recommendations for families and professionals to strengthen implementation, broaden perspectives, and improve outcomes for Deaf children.
